# Clinicopathological characteristics and eligibility for adjuvant olaparib of germline *BRCA1/2* mutation carriers with HER2-negative early breast cancer

**DOI:** 10.1038/s41523-024-00632-8

**Published:** 2024-04-16

**Authors:** Stefania Morganti, Qingchun Jin, Julie Vincuilla, Ryan Buehler, Sean Ryan, Samantha Stokes, Tonia Parker, Elizabeth A. Mittendorf, Tari A. King, Anna Weiss, Ann H. Partridge, Brittany L. Bychkovsky, Giuseppe Curigliano, Nabihah Tayob, Nancy U. Lin, Judy E. Garber, Sara M. Tolaney, Filipa Lynce

**Affiliations:** 1 Department of Medical Oncology, Dana-Farber Cancer Institute, Boston, MA USA; 2 Breast Oncology Program, Dana-Farber Brigham Cancer Center, Boston, MA USA; 3 Harvard Medical School, Boston, MA USA; 4 Broad Institute of MIT and Harvard, Boston, MA USA; 5 Department of Data Science, Dana-Farber Cancer Institute, Boston, MA USA; 6 Division of Cancer Genetics and Prevention, Dana-Farber Cancer Institute, Boston, MA USA; 7 Division of Breast Surgery, Department of Surgery, Brigham and Women’s Hospital, Boston, MA USA; 8 Department of Oncology and Hemato-Oncology, University of Milan, Milan, Italy; 9 European Institute of Oncology IRCCS, Milan, Italy; 10 Present Address: Division of Surgical Oncology, University of Rochester Medical Center, Rochester, NY USA

**Keywords:** Breast cancer, Cancer therapy

## Abstract

Following the survival benefit demonstrated in the OlympiA trial, one year of adjuvant olaparib is now recommended for all patients with germline *BRCA1/2* pathogenic/likely pathogenic variants (PV) and high-risk, HER2-negative early breast cancer after chemotherapy. However, optimal identification of high-risk patients who may derive benefit from this genomically-directed therapy is debated. In this study, we sought to characterize the real-world proportion of *gBRCA1/2* PV carriers eligible for adjuvant olaparib according to the OlympiA criteria, and to compare clinicopathologic characteristics and outcomes between eligible and ineligible patients.

Approximately 5% of breast cancers occur in patients who carry a germline *BRCA1/2* (*gBRCA1/2*) pathogenic or likely pathogenic variant (PV)^1,2^. In the OlympiA trial one year of adjuvant olaparib improved invasive disease-free survival (iDFS) and overall survival (OS) in g*BRCA1/2* PV carriers with high-risk, HER2-negative early breast cancer^3,4^, and olaparib became the first systemic adjuvant therapy specifically approved for these patients. Eligibility criteria of OlympiA differed for triple-negative breast cancer (TNBC) and hormone receptor-positive (HR+) tumors. Patients with TNBC were eligible either when residual disease was present after neoadjuvant chemotherapy, or after upfront surgery and adjuvant chemotherapy for tumors ≥2 cm or with nodal involvement. Patients with HR+ tumors were eligible either if there was residual disease and a clinical and pathologic stage (CPS) and estrogen receptor status and histologic grade (EG) (CPS + EG) score ≥3 after neoadjuvant chemotherapy, or if ≥4 nodes were involved at surgery prior to adjuvant chemotherapy.

Whether these criteria identify all gBRCA PV carriers with high-risk breast tumors who may benefit from olaparib is debated. This is particularly relevant for HR+ breast cancers, as alternative trials used different criteria to select high-risk patients. In monarchE, for instance, patients were eligible either if they had ≥4 positive nodes at surgery, or 1-3 positive nodes and at least one additional high-risk criterion among grade 3 disease, tumor size > 5 cm, or Ki67 ≥ 20%. In this study, we used a prospectively maintained single institution database to characterize the real-world proportion of *gBRCA1/2* PV carriers with early breast cancer who meet OlympiA inclusion criteria, and compared clinicopathologic characteristics and outcomes between eligible and ineligible patients. Additionally, we investigated the overlap between criteria in OlympiA and monarchE in an effort to identify additional high-risk patients who might benefit from novel targeted therapies in the adjuvant setting.

We identified 205 g*BRCA1/2* PV carriers with newly diagnosed, HER2-negative early breast cancer, including 113 with HR+ and 92 with TNBC (Fig. 1, Supplementary Table 1). Of them, 15 had synchronous primaries for which only the highest risk tumor was considered in the analysis. Median age at diagnosis was 43 years and most patients (*n* = 129, 62.9%) were premenopausal. A g*BRCA1* PV was identified in 115 (56.1%) patients and a g*BRCA2* PV in 90 (43.9%). Overall, 73 (35.6%) patients underwent genetic testing before the diagnosis of breast cancer. A total of 166 (81.0%) patients received chemotherapy; of them, 130 (78.3%) received an anthracycline-containing regimen and 32 (19.3%) received platinum. Neoadjuvant chemotherapy was administered to 107 patients (77 *gBRCA1*, 30 *gBRCA2*), of whom 47 (40 *gBRCA1*, 7 *gBRCA2*) achieved a pCR (43.9%). Only 8 (3.9%) patients received immunotherapy. Eleven patients received (neo)adjuvant PARP inhibitors, and 4 participated in the OlympiA trial.

**Fig. 1:**
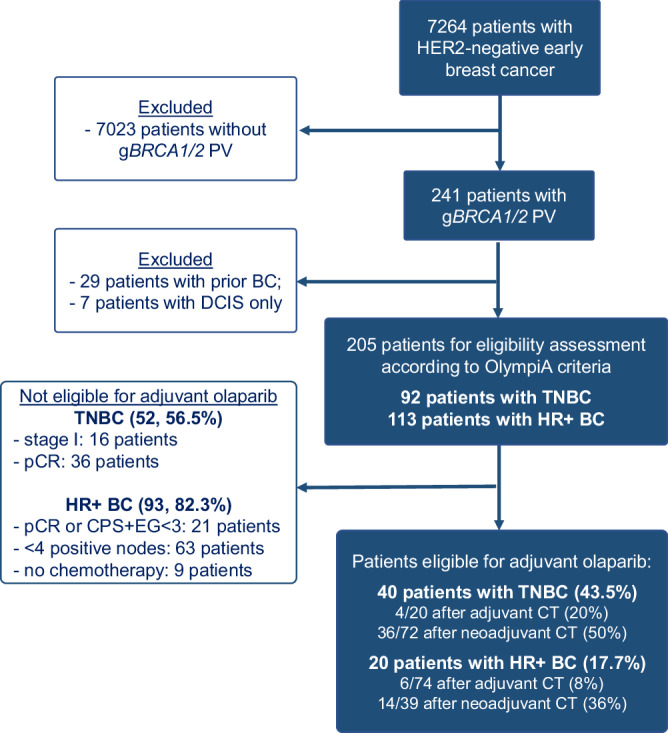
STROBE diagram*.* BC breast cancer, DCIS ductal carcinoma in situ, gBRCA PV germline BRCA pathogenic or likely pathogenic variant, HER2 human epidermal growth factor receptor 2, HR+ hormone receptor positive, pCR pathologic complete response, TNBC triple-negative breast cancer.

Overall, 60 (29.3%) patients were eligible for adjuvant olaparib according to OlympiA criteria (39 g*BRCA1* and 21 g*BRCA2* PV carriers), including 40 (66.7%) with TNBC and 20 (33.3%) with HR+ breast cancer (Table 1; Fig. 2).

**Table 1:** Comparison of clinicopathological characteristics and treatments between eligible and ineligible patients

Characteristics	Overall (*N* = 205)	OlympiA eligible (*N* = 60)	OlympiA ineligible (*N* = 145)	*P* value
**Age at diagnosis**				
Mean (SD)	45.9 (13.6)	43.5 (12.8)	46.9 (13.8)	0.094
Median [Min, Max]	43.0 [25.0, 80.0]	39.0 [25.0, 73.0]	44.0 [25.0, 80.0]	
**Sex**				
Female	198 (96.6%)	58 (96.7%)	140 (96.6%)	1
Male	7 (3.4%)	2 (3.3%)	5 (3.4%)	
**Race**				
White	176 (85.9%)	51 (85.0%)	125 (86.2%)	0.600
Asian or Pacific Islander	9 (4.4%)	2 (3.3%)	7 (4.8%)	
African American	10 (4.9%)	2 (3.3%)	8 (5.5%)	
Native American, Alaska Natives	1 (0.5%)	0 (0%)	1 (0.7%)	
Unknown	9 (4.4%)	5 (8.3%)	4 (2.8%)	
**Menopausal status**				
Postmenopausal	67 (32.7%)	17 (28.3%)	50 (34.5%)	0.443
Premenopausal	129 (62.9%)	41 (68.3%)	88 (60.7%)	
Unknown	9 (4.4%)	2 (3.3%)	7 (4.8%)	
**Gene**				
*BRCA1*	115 (56.1%)	39 (65.0%)	76 (52.4%)	0.134
*BRCA2*	90 (43.9%)	21 (35.0%)	69 (47.6%)	
**Clinical presentation**				
Screen-detected	76 (37.1%)	14 (23.3%)	62 (42.8%)	**0.026**
Breast lump	116 (56.5%)	41 (68.4%)	75 (51.8%)	
Other breast/axillary symptoms	13 (6.3%)	5 (8.3%)	8 (5.5%)	
**Tumor histology**				
Invasive ductal	165 (80.5%)	48 (80.0%)	117 (80.7%)	1
Invasive lobular	16 (7.8%)	5 (8.3%)	11 (7.6%)	
Mixed (IDC & ILC)	20 (9.8%)	6 (10.0%)	14 (9.7%)	
Other	4 (1.9%)	1 (1.7%)	3 (2.1%)	
**Synchronous breast cancer**				
Yes	15 (7.3%)	4 (6.7%)	11 (7.6%)	1
No	190 (92.7%)	56 (93.3%)	134 (92.4%)	
**Tumor Grade**				
I Low Grade	7 (3.4%)	0 (0%)	7 (4.8%)	**0.046**
II Intermediate Grade	48 (23.4%)	10 (16.7%)	38 (26.2%)	
III High Grade	148 (72.2%)	50 (83.3%)	98 (67.6%)	
Unknown	2 (1.0%)	0 (0%)	2 (1.4%)	
**Subtype**				
HR+	113 (55.1%)	20 (33.3%)	93 (64.1%)	<**0.001 **
HR-low (ER < 10% and/or PR < 10%)	15 (13.3%)	6 (30.0%)	9 (9.7%)	
TN	92 (44.9%)	40 (66.7%)	52 (35.9%)	
**HER2 IHC score**				
0	94 (45.9%)	31 (51.7%)	63 (43.4%)	0.188
1+	66 (32.2%)	20 (33.3%)	46 (31.7%)	
2+ (with negative FISH)	40 (19.5%)	7 (11.7%)	33 (22.8%)	
Not done/Unknown	5 (2.4%)	2 (3.3%)	3 (2.1%)	
**Stage**				
I	83 (40.5%)	5 (8.3%)	78 (53.8%)	<**0.001**
II	93 (45.4%)	38 (63.3%)	55 (37.9%)	
III	29 (14.1%)	17 (28.3%)	12 (8.3%)	
**Chemotherapy**				
Yes	166 (81.0%)	60 (100%)	106 (73.1%)	**<0.001**
Adjuvant only	59 (28.8%)	10 (16.7%)	49 (33.8%)	
Neoadjuvant only	56 (27.3%)	12 (20.0%)	44 (30.3%)	
Both neoadjuvant and adjuvant	51 (24.9%)	38 (63.3%)	13 (9.0%)	
No	39 (19.0%)	0 (0%)	39 (26.9%)	
**Received anthracyclines**				
Yes	130 (78.3%)	55 (91.7%)	75 (70.8%)	**0.002**
No	36 (21.7%)	5 (8.3%)	31 (29.2%)	
**Received platinum**				
Yes	32 (19.3%)	19 (31.7%)	13 (12.3%)	**0.005**
No	134 (80.7%)	41 (68.3%)	93 (87.7%)	
**Endocrine therapy (Among HR+ Patients)**				
Yes	91 (80.5%)	12 (60%)	79 (84.9%)	**0.025**
No	22 (19.5%)	8 (40%)	14 (15.1%)	
**ODX performed? (Among HR+ Patients)**				
Performed	50 (44.2%)	2 (10%)	48 (51.6%)	**0.001**
Not performed	63 (55.8%)	18 (90%)	45 (48.4%)	
**ODX Recurrence Score**				
Median [Min, Max]	25.0 [10.0, 71.0]	32.5 [30.0, 35.0]	25.0 [10.0, 71.0]	–
**Genetic testing before diagnosis**				
Yes	73 (35.6%)	12 (20.0%)	61 (42.1%)	**0.004**
No	132 (64.4%)	48 (80.0%)	84 (57.9%)	

*FISH* fluorescence in situ hybridization, *HER2* human epidermal growth factor receptor 2, *HR* hormone receptor, *IDC* invasive ductal carcinoma, *IHC* immunohistochemistry, *ILC* invasive lobular carcinoma, *ODX* Oncotype Dx, *SD* standard deviation, *TN* triple negative. Significant *P* values (*P* < 0.05) are highlighted in bold.

**Fig. 2:**
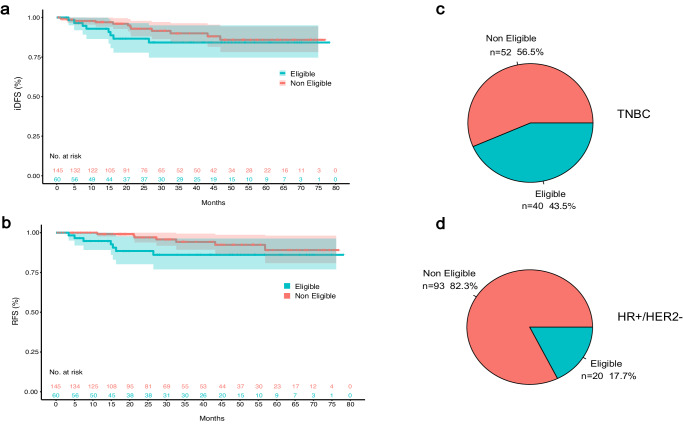
Prevalence and outcomes of *gBRCA1/2* pathogenic/likely pathogenic variants carriers according to eligibility for adjuvant olaparib. **a** iDFS for patients eligible and ineligible for adjuvant olaparib; **b** RFS for patients eligible and ineligible for adjuvant olaparib; **c** Proportion of eligible and ineligible patients among gBRCA PV carriers with TNBC; **d** Proportion of eligible and ineligible patients among gBRCA PV carriers with HR+/HER2− breast cancer. iDFS invasive disease-free survival, RFS recurrence-free survival, HER2− human epidermal growth factor receptor 2 negative, HR+ hormone receptor positive, PV pathogenic/likely pathogenic, TNBC triple-negative breast cancer.

Major reasons why most patients identified with g*BRCA1*/2 PV and breast cancer were deemed ineligible for adjuvant olaparib were lack of prior chemotherapy (*n* = 9), low anatomic stage at upfront surgery (*n* = 16 with stage I TNBC; *n* = 63 with HR+ tumors and <4+ nodes), or evidence of substantial response to neoadjuvant chemotherapy (*n* = 36 TNBC had pCR; *n* = 21 with HR+ disease had a pCR or a CPS&EG score <3).

Eligible patients were more likely to have higher grade and higher stage tumors (*p* < 0.001) compared to ineligible patients. Consistent with the higher stage at presentation, eligible patients were less frequently diagnosed through screening imaging (23.3 vs 42.8%, *p* = 0.026) and had genetic testing performed more often after diagnosis (80 vs 57.9%, *p* = 0.004). Chemotherapy regimens administered to eligible patients more frequently included anthracyclines (*p* = 0.002) and/or platinum salts (*p* = 0.005). Among patients with HR+ breast cancer, only 2 out of 20 (10%) patients eligible for adjuvant olaparib had recurrence scores (RS) assessed, compared to 48 out of 93 (51.6%) ineligible patients (Table 1).

After a median follow up for disease status of 31 months (IQR 16.53 months), 20 iDFS and 13 recurrence-free survival (RFS) events were recorded (Table 2). Three-year iDFS was 84.2% (95% CI, 74.6–95.0%) for eligible and 90.0% (95% CI, 84.1–96.3%) for ineligible patients (hazard ratio 1.54 [95% CI, 0.63–3.78], *p* = 0.34). Three-year RFS was 86.1% (95% CI, 77.0–96.3%) for eligible and 94.2% (95% CI, 89.3–99.4%) for ineligible patients (hazard ratio 2.38 [95% CI, 0.83–6.80], *p* = 0.11) (Fig. 2).

**Table 2:** Invasive disease-free survival (iDFS) events

First iDFS events (20)	Eligible patients (8)	Ineligible patients (12)
Locoregional recurrence (4)	1	3
Distant recurrence (9)	6	3
Second primary malignancy^a^ (7)	1	6

^a^Eligible: tubal carcinoma; ineligible: ovarian cancer (2), thyroid cancer, pancreatic cancer, contralateral breast cancer, lung carcinoid.

Similar results were observed when comparing eligible versus ineligible patients among g*BRCA1* and g*BRCA2* PVs separately, and among patients with HR+ disease (Table 3). Interestingly, among patients with TNBC, eligible patients had substantially worse outcomes compared to those who were ineligible, with a 12.8% and 12.1% 3-year iDFS and RFS absolute difference, respectively, although with a small number of events and large confidence intervals. Overall, outcomes appeared to be worse for g*BRCA2*-associated breast tumors compared to g*BRCA1*, and for HR+ tumors compared to TNBC (Table 3).

**Table 3:** Subgroup analyses of invasive disease-free survival and recurrence-free survival

Characteristic	Number of events	% 3-year iDFS (95% CI)	Hazard ratio (95% CI), *p* value
**All patients (*n*** = **205)**	18	88.3 (83.2–93.7)	
Eligible	8	84.2 (74.6–95.0)	eligible vs ineligible: HR 1.54 (0.63–3.78), *p* = 0.34
Ineligible	10	90.0 (84.1–96.3)	
*BRCA1*	6	92.7 (87.0–98.8)	*BRCA1* vs *BRCA2*: HR 0.42 (0.17–1.06), *p* = 0.07
*BRCA2*	12	83.1 (74.6–92.4)	
HR+	12	85.3 (77.9–93.5)	HR+ vs TNBC: HR 1.55 (0.62–3.89), *p* = 0.35
TNBC	6	92.1 (86.1–98.6)	
***BRCA1*** **(*n*** = **115)**			
Eligible	3	90.6 (80.8–100.0)	eligible vs ineligible: HR 1.57 (0.35–7.05), *p* = 0.55
Ineligible	3	93.8 (86.8–100.0)	
***BRCA2*** **(*n*** = **90)**			
Eligible	5	76.2 (60.0–96.8)	eligible vs ineligible: HR 1.63 (0.53–5.00), *p* = 0.39
Ineligible	7	85.6 (76.0–96.4)	
**HR** + **(*n*** = **113)**			
Eligible	3	82.6 (66.6–100.0)	eligible vs ineligible: HR 1.28 (0.35–4.66), *p* = 0.71
Ineligible	9	85.7 (77.2–95.1)	
**TNBC (*n*** = **92)**			
Eligible	5	85.2 (73.7–98.4)	eligible vs ineligible: HR 3.1 (0.60–16.02), *p* = 0.18
Ineligible	1	98.0 (94.3–100.0)	

Characteristic	Number of events	% 3-year RFS (95% CI)	Hazard ratio (95% CI), *p* value
**All patients (*n*** = **205)**	12	91.8 (87.4–96.5)	
Eligible	7	86.1 (77.0–96.3)	eligible vs ineligible: HR 2.38 (0.83–6.8), *p* = 0.11
Ineligible	5	94.2 (89.3–99.4)	
*BRCA1*	4	94.6 (89.4–100.0)	*BRCA1* vs *BRCA2*: HR 0.33 (0.10–1.04), *p* = 0.06
*BRCA2*	8	88.5 (81.2–96.4)	
HR+	8	89.8 (83.3–96.9)	HR+ vs TNBC: HR 2.05 (0.64–6.53), *p* = 0.23
TNBC	4	94.5 (89.2–100.0)	
***BRCA1*** **(*n*** = **115)**			
Eligible	2	93.6 (85.3–100.0)	eligible vs ineligible: HR 2.17 (0.30–15.4), *p* = 0.44
Ineligible	2	95.1 (88.6–100.0)	
***BRCA2*** **(*n*** = **90)**			
Eligible	5	76.2 (60.0–96.8)	eligible vs ineligible: HR 2.78 (0.80–9.64), *p* = 0.11
Ineligible	3	93.3 (86.1–100.0)	
**HR** + **(*n*** = **113)**			
Eligible	3	82.6 (66.6–100.0)	eligible vs ineligible: HR 1.92 (0.50–7.47), *p* = 0.34
Ineligible	5	91.3 (84.1–99.0)	
**TNBC (*n*** = **92)**			
Eligible	4	87.9 (77.3–100.0)	eligible vs ineligible: HR NA, ***p*** = **0.03**
Ineligible	0	100.0 (100.0–100.0)	

*CI* confidence interval, *HR+* hormone receptor-positive, *HR* hazard ratio, *iDFS* invasive disease-free survival, *NA* not applicable, *RFS* recurrence-free survival, *TNBC* triple-negative breast cancer. Significant *p* values (*p* < 0.05) are highlighted in bold.

Of the 113 patients with HR+ breast cancer, 16 (14%) were eligible for both adjuvant olaparib and abemaciclib (2 received abemaciclib, 1 received both agents, 1 enrolled in OlympiA), 4 (3%) patients were eligible for olaparib only (1 received olaparib and 1 enrolled in OlympiA) and 18 (8.8%) for abemaciclib only (3 received abemaciclib). Using monarchE criteria, we identified 18 (8.8%) additional patients who may be considered for treatment escalation who were not identified by OlympiA criteria.

In this study, we analyzed a large cohort of g*BRCA1/2* PV carriers to assess the proportion of patients in a real-world setting deemed eligible for adjuvant olaparib according to OlympiA criteria. Among our patients, approximately 30% met OlympiA criteria. When comparing clinicopathologic characteristics between eligible and ineligible patients, we observed that eligible patients were less frequently diagnosed following imaging-based screening and more often underwent genetic testing after diagnosis. Although this is expected given OlympiA’s requirements in terms of tumor stage at diagnosis, it also underlines that earlier recognition of g*BRCA1/2* PV carriers with associated recommended breast imaging monitoring could allow identification of smaller tumors with higher chance of cure following primary therapy.

We observed a large number of RFS events among ineligible patients although, as expected, there was a non-significant trend towards worse outcomes for eligible compared to ineligible patients. Similar iDFS and RFS differences were observed among eligible and ineligible patients when g*BRCA1* PV carriers, g*BRCA2* PV carriers, and patients with HR+ or TNBC tumors were analyzed separately. Interestingly, the gap between eligible and ineligible patients appeared larger for TNBC, which may suggest that the eligibility criteria employed in OlympiA were better at discriminating between high- and low-risk for TNBC than for HR+ disease. Of note, when comparing monarchE and OlympiA inclusion criteria, we observed that almost half of high-risk patients defined by monarchE eligibility criteria did not meet OlympiA criteria.

This present analysis has limitations inherent to the nature of this study. First, we used real-world data, and survival measures may be biased by how patients were monitored over time. Second, the small sample size and relatively low number of events limits the power of our analyses, in particular for subgroup analyses. Third, we did not consider multifocal disease, which is common among *gBRCA1/*2 PV carriers and does impact risk of recurrence. Fourth, our median follow up of 31 months is relatively short, especially given the known long-term risk of relapse of HR+ breast tumors. Fifth, 35.6% of patients underwent genetic testing before receiving the diagnosis of breast cancer, which may have favored the detection of lower stage tumors and reduced the proportion of patients eligible for adjuvant olaparib.

To date, olaparib is recommended for all patients with g*BRCA1/2* PV and HER2-negative breast cancer considered at high-risk of recurrence, although the definition of “high-risk” remains unclear^5^. It is worth noticing that both the U.S. Food and Drug Administration^6^ and the European Medical Agency^7^ approved adjuvant olaparib for gBRCA PV carriers with high-risk, early-stage, HER2-negative breast cancer, without considering the specifics of OlympiA criteria. Here, in a real world-cohort of patients with gBRCA PV, we described that only 30% met the OlympiA eligibility criteria, and that ineligible patients were still at high risk of recurrence, especially those with HR+ tumors. Therefore, the proportion of gBRCA PV carriers who may benefit from adjuvant olaparib is likely to extend beyond OlympiA criteria. This choice allowed the study to report results sooner, but likely excluded lower risk patients who may benefit from this approach. However, exactly how broad eligibility should be is unknown, and further research is needed to understand whether low risk patients could also benefit from adjuvant PARP inhibitors. In this setting, whether these agents could replace chemotherapy is worth of further investigation.

## Methods

### Study design

Clinicopathologic and genetic data from all consecutive patients with *gBRCA1/2* PV who underwent surgery between 1/4/2016 and 4/7/2022 for a first diagnosis of HER2-negative invasive breast cancer at Dana-Farber Brigham Cancer Center between 2016 and 2022 were extracted from prospectively collected institutional datasets. HR+ disease was defined according to ASCO/CAP guidelines (i.e., estrogen and/or progesterone receptor ≥1%). Patients with prior invasive breast cancer were excluded, whereas patients with prior invasive non-breast cancer were included in the analysis. For patients with synchronous breast tumors, the highest risk tumor was considered. Eligibility for adjuvant olaparib was defined according to the OlympiA study inclusion criteria^4^. This study was conducted in accordance with the Declaration of Helsinki. The Dana-Farber/Harvard Cancer Center Institutional Review Board classified this study as exempt from IRB approval and included a waiver of informed consent in accordance with the U.S Common Rule.

### Statistical analysis

Clinicopathological characteristics and treatment patterns were compared between eligible and ineligible patients using Fisher’s exact test, Chi-squared test, or Wilcoxon rank-sum test, as appropriate. iDFS and RFS were defined according to STEEP 2.0 criteria^8^ and calculated using the Kaplan-Meier method with the log-rank test to compare between eligible and ineligible patients. As exploratory analyses, the proportion of gBRCA PV carriers with HR+ breast cancer deemed as high-risk by the monarchE criteria (1–3 positive nodes and grade 3 or tumor size >5 cm)^9^ and the overlap between eligibility to OlympiA and monarchE were assessed. All statistical tests were two-sided with *P* values ≤ 0.05 considered statistically significant.

### Supplementary information

Supplementary Table 1

Related Manuscript File

## Data Availability

The data that support the findings of this study are available from the corresponding author upon reasonable request.

## Code availability

All analyses were performed with R version 4.0.3. R codes are available from the corresponding author on reasonable request.
