# Supplementary material for: Clinicopathological characteristics and eligibility for adjuvant olaparib of germline BRCA1/2 mutation carriers with HER2-negative early breast cancer
Source: NPJ Breast Cancer. 2024 Apr 16;10:28. doi: 10.1038/s41523-024-00632-8 (PMC11021554; doi:10.1038/s41523-024-00632-8)
Supplement: Supplementary file 1 — Supplementary Table 1 [file 41523_2024_632_MOESM1_ESM.pdf]

Supplementary Table 1. g*BRCA1/2* variants

ID	Gene	Classification	Alteration
1	*BRCA1*	Pathogenic	1135insA
2	*BRCA1*	Pathogenic	187delAG
3	*BRCA1*	Pathogenic	187delAG
4	*BRCA1*	Pathogenic	187delAG
5	*BRCA1*	Pathogenic	187delAG
6	*BRCA1*	Pathogenic	187delAG
7	*BRCA1*	Pathogenic	187delAG
8	*BRCA1*	Pathogenic	2826insAT
9	*BRCA1*	Pathogenic	3450del4
10	*BRCA1*	Pathogenic	4184del4
11	*BRCA1*	Pathogenic	4603G>T
12	*BRCA1*	Pathogenic	5266dupC
13	*BRCA1*	Pathogenic	5382insC
14	*BRCA1*	Pathogenic	5385insC
15	*BRCA1*	Pathogenic	5385insC
16	*BRCA1*	Pathogenic	c.116G>A
17	*BRCA1*	Pathogenic	c.1175_1214del40
18	*BRCA1*	Pathogenic	c.130T>A
19	*BRCA1*	Pathogenic	c.135-1G>T
20	*BRCA1*	Pathogenic	c.135-1G>T
21	*BRCA1*	Pathogenic	c.1687C>T
22	*BRCA1*	Pathogenic	c.1687C>T
23	*BRCA1*	Pathogenic	c.181T>G
24	*BRCA1*	Pathogenic	c.181T>G
25	*BRCA1*	Pathogenic	c.1823_1826delAGAA
26	*BRCA1*	Pathogenic	c.1953_1956delGAAA
27	*BRCA1*	Pathogenic	c.212+3A>G
28	*BRCA1*	pathogenic	c.213-11T>G
29	*BRCA1*	Pathogenic	c.213-12A>G
30	*BRCA1*	Pathogenic	c.2329delT
31	*BRCA1*	Pathogenic	c.2389G>T
32	*BRCA1*	Pathogenic	c.2411_2412delAG
33	*BRCA1*	Pathogenic	c.2685_2686del
34	*BRCA1*	Pathogenic	c.2835_2836insCC
35	*BRCA1*	Pathogenic	c.2835_2836insCC
36	*BRCA1*	Pathogenic	c.2940delA
37	*BRCA1*	Pathogenic	c.3331_3334delCAAG
38	*BRCA1*	Pathogenic	c.3436_3439delTGTT
39	*BRCA1*	Pathogenic	c.3734del
40	*BRCA1*	Pathogenic	c.4035del

41	*BRCA1*	Pathogenic	c.4035del
42	*BRCA1*	Pathogenic	c.4035del
43	*BRCA1*	Pathogenic	c.4065_4068del
44	*BRCA1*	Pathogenic	c.4065_4068del
45	*BRCA1*	Pathogenic	c.4065_4068delTCAA
46	*BRCA1*	Pathogenic	c.4096+1G>A
47	*BRCA1*	Pathogenic	c.4183C>T
48	*BRCA1*	Pathogenic	c.4327C>T
49	*BRCA1*	Pathogenic	c.4357+1G>A
50	*BRCA1*	Pathogenic	c.4391_4393delinsTT
51	*BRCA1*	Pathogenic	c.4459A>T
52	*BRCA1*	Pathogenic	c.4484+1G>A
53	*BRCA1*	Pathogenic	c.4484G>T
54	*BRCA1*	Pathogenic	c.4689C>G
55	*BRCA1*	Pathogenic	c.4689C>G
56	*BRCA1*	Pathogenic	c.4689C>G
57	*BRCA1*	Pathogenic	c.4964_4982del
58	*BRCA1*	Pathogenic	c.4964_4982del19
59	*BRCA1*	Pathogenic	c.4986+6T>C
60	*BRCA1*	Pathogenic	c.5035_5039del
61	*BRCA1*	Pathogenic	c.5035del
62	*BRCA1*	Pathogenic	c.5095C>T
63	*BRCA1*	Pathogenic	c.5096G>A
64	*BRCA1*	Pathogenic	c.5216A>G
65	*BRCA1*	Likely Pathogenic	c.5242G>T
66	*BRCA1*	Pathogenic	c.5251C>T
67	*BRCA1*	Pathogenic	c.5266dup
68	*BRCA1*	Pathogenic	c.5266dup
69	*BRCA1*	Pathogenic	c.5266dup
70	*BRCA1*	Pathogenic	c.5266dup
71	*BRCA1*	Pathogenic	c.5266dupC
72	*BRCA1*	Pathogenic	c.5266dupC
73	*BRCA1*	Pathogenic	c.5266dupC
74	*BRCA1*	Pathogenic	c.5266dupC
75	*BRCA1*	Pathogenic	c.5266dupC
76	*BRCA1*	Pathogenic	c.5266dupC
77	*BRCA1*	Pathogenic	c.679G>T
78	*BRCA1*	Pathogenic	c.68_69del
79	*BRCA1*	Pathogenic	c.68_69del
80	*BRCA1*	Pathogenic	c.68_69del
81	*BRCA1*	Pathogenic	c.68_69delAG
82	*BRCA1*	Pathogenic	c.68_69delAG
83	*BRCA1*	Pathogenic	c.68_69delAG

84	*BRCA1*	Pathogenic	c.68_69delAG
85	*BRCA1*	Pathogenic	c.68_69delAG
86	*BRCA1*	Pathogenic	c.68_69delAG
87	*BRCA1*	Pathogenic	c.68_69delAG
88	*BRCA1*	Pathogenic	c.68_69delAG
89	*BRCA1*	Pathogenic	c.815_824dup
90	*BRCA1*	Pathogenic	c.815_824dup10
91	*BRCA1*	Pathogenic	c.844_850dupTCATTAC
92	*BRCA1*	Pathogenic	c.933del
93	*BRCA1*	Pathogenic	c.962G>A
94	*BRCA1*	Pathogenic	c.981_982del
95	*BRCA1*	Pathogenic	del exon 15
96	*BRCA1*	Pathogenic	del exon 3
97	*BRCA1*	Pathogenic	del exons 1-23
98	*BRCA1*	Pathogenic	del exons 14-19
99	*BRCA1*	Pathogenic	del exons 20-23
100	*BRCA1*	Pathogenic	dupl exon 12, copy number=3
101	*BRCA1*	Pathogenic	EX11del
102	*BRCA1*	Pathogenic	EX13_14del
103	*BRCA1*	Pathogenic	EX2del
104	*BRCA1*	Pathogenic	specific alteration not available
105	*BRCA1*	Pathogenic	specific alteration not available
106	*BRCA1*	Pathogenic	specific alteration not available
107	*BRCA1*	Pathogenic	specific alteration not available
108	*BRCA1*	Pathogenic	specific alteration not available
109	*BRCA1*	Pathogenic	specific alteration not available
110	*BRCA1*	Pathogenic	specific alteration not available
111	*BRCA1*	Pathogenic	specific alteration not available
112	*BRCA1*	Pathogenic	specific alteration not available
113	*BRCA1*	Pathogenic	specific alteration not available
114	*BRCA1*	Pathogenic	specific alteration not available
115	*BRCA1*	Pathogenic	specific alteration not available
116	*BRCA2*	Pathogenic	5909insA
117	*BRCA2*	Pathogenic	6174delT
118	*BRCA2*	Pathogenic	8765delAG
119	*BRCA2*	Pathogenic	8765delAG
120	*BRCA2*	Pathogenic	8765delAG
121	*BRCA2*	Pathogenic	886delGT
122	*BRCA2*	Pathogenic	c.100G>T
123	*BRCA2*	Pathogenic	c.10152C>G
124	*BRCA2*	Pathogenic	c.1029del
125	*BRCA2*	Pathogenic	c.1172del
126	*BRCA2*	Pathogenic	c.1189_1190insTTAG

127	*BRCA2*	Pathogenic	c.1310_1313del
128	*BRCA2*	Pathogenic	c.1310_1313del
129	*BRCA2*	Pathogenic	c.1310_1313del
130	*BRCA2*	Pathogenic	c.1681G>T
131	*BRCA2*	Pathogenic	c.1813dupA
132	*BRCA2*	Pathogenic	c.1889delC
133	*BRCA2*	Pathogenic	c.1929del
134	*BRCA2*	Pathogenic	c.2330dup
135	*BRCA2*	Pathogenic	c.2731del
136	*BRCA2*	Pathogenic	c.2808_2811delACAA
137	*BRCA2*	Pathogenic	c.3083_3084ins(?)
138	*BRCA2*	Pathogenic	c.3170_3174del
139	*BRCA2*	Pathogenic	c.3385C>T
140	*BRCA2*	Pathogenic	c.3847_3848del
141	*BRCA2*	Pathogenic	c.3922G>T
142	*BRCA2*	Pathogenic	c.4058_4062delAAACG
143	*BRCA2*	Pathogenic	c.407delA
144	*BRCA2*	Pathogenic	c.4563_4564delAT
145	*BRCA2*	Pathogenic	c.4829_4830delTG
146	*BRCA2*	Pathogenic	c.5073dupA
147	*BRCA2*	Pathogenic	c.5241_5242insTA
148	*BRCA2*	Pathogenic	c.5271T>A
149	*BRCA2*	Pathogenic	c.5350_5351del
150	*BRCA2*	Pathogenic	c.5350_5351delAA
151	*BRCA2*	Pathogenic	c.5576_5579del
152	*BRCA2*	Pathogenic	c.5576_5579delTTAA
153	*BRCA2*	Pathogenic	c.5682C>A
154	*BRCA2*	Pathogenic	c.5722_5723del
155	*BRCA2*	Pathogenic	c.5828del
156	*BRCA2*	Pathogenic	c.5857G>T
157	*BRCA2*	Pathogenic	c.5946del
158	*BRCA2*	Pathogenic	c.5946del
159	*BRCA2*	Pathogenic	c.5946del
160	*BRCA2*	Pathogenic	c.5946del
161	*BRCA2*	Pathogenic	c.5946delT
162	*BRCA2*	Pathogenic	c.5946delT
163	*BRCA2*	Pathogenic	c.5946delT
164	*BRCA2*	Pathogenic	c.5946delT
165	*BRCA2*	Pathogenic	c.5946delT
166	*BRCA2*	Pathogenic	c.5946delT
167	*BRCA2*	Pathogenic	c.5946delT
168	*BRCA2*	Pathogenic	c.5946delT
169	*BRCA2*	Pathogenic	c.6079dup

170	*BRCA2*	Pathogenic	c.6275_6276delTT
171	*BRCA2*	Pathogenic	c.6450dupA
172	*BRCA2*	Pathogenic	c.6486_6489del
173	*BRCA2*	Pathogenic	c.658_659del
174	*BRCA2*	Pathogenic	c.658_659delGT
175	*BRCA2*	Pathogenic	c.6591_6592del
176	*BRCA2*	Pathogenic	c.6644_6647delACTC
177	*BRCA2*	Likely Pathogenic	c.682-1G>C
178	*BRCA2*	Pathogenic	c.7007G>A
179	*BRCA2*	Pathogenic	c.7252C>T
180	*BRCA2*	Pathogenic	c.7558C>T
181	*BRCA2*	Pathogenic	c.7558C>T
182	*BRCA2*	Pathogenic	c.771_775delTCAAA
183	*BRCA2*	Pathogenic	c.7762_7764delinsTT
184	*BRCA2*	Pathogenic	c.7940T>C
185	*BRCA2*	Pathogenic	c.7958T>C
186	*BRCA2*	Pathogenic	c.7977-1G>C
187	*BRCA2*	Pathogenic	c.8487+1G>A
188	*BRCA2*	Likely Pathogenic	c.8487+3A>G
189	*BRCA2*	Pathogenic	c.8488-1G>A
190	*BRCA2*	Pathogenic	c.8537_8538del
191	*BRCA2*	Pathogenic	c.8537_8538delAG
192	*BRCA2*	Pathogenic	c.8607delT
193	*BRCA2*	Pathogenic	c.8904delC
194	*BRCA2*	Pathogenic	c.9004G>A
195	*BRCA2*	Pathogenic	c.9253delA
196	*BRCA2*	Pathogenic	c.9253dupA
197	*BRCA2*	Pathogenic	c.9382 C>T
198	*BRCA2*	Likely Pathogenic	c.9648+1G>C
199	*BRCA2*	Pathogenic	c.9924C>G
200	*BRCA2*	Pathogenic	specific alteration not available
201	*BRCA2*	Pathogenic	specific alteration not available
202	*BRCA2*	Pathogenic	specific alteration not available
203	*BRCA2*	Pathogenic	specific alteration not available
204	*BRCA2*	Pathogenic	specific alteration not available
205	*BRCA2*	Pathogenic	specific alteration not available
