# Supplementary material for: Clinicopathological characteristics and eligibility for adjuvant olaparib of germline BRCA1/2 mutation carriers with HER2-negative early breast cancer
Source: NPJ Breast Cancer. 2024 Apr 16;10:28. doi: 10.1038/s41523-024-00632-8 (PMC11021554; doi:10.1038/s41523-024-00632-8)
Supplement: Supplementary file 2 — Related Manuscript File [file 41523_2024_632_MOESM2_ESM.pdf]

nature portfolio

Corresponding author(s): Filipa Lynce

Last updated by author(s): Mar 14, 2024

# Reporting Summary

Nature Portfolio wishes to improve the reproducibility of the work that we publish. This form provides structure for consistency and transparency in reporting. For further information on Nature Portfolio policies, see our Editorial Policies and the Editorial Policy Checklist.

## Statistics

For all statistical analyses, confirm that the following items are present in the figure legend, table legend, main text, or Methods section.

| n/a | Confirmed | |
|---|---|---|
| ☐ | ☒ | The exact sample size (*n*) for each experimental group/condition, given as a discrete number and unit of measurement |
| ☐ | ☒ | A statement on whether measurements were taken from distinct samples or whether the same sample was measured repeatedly |
| ☐ | ☒ | The statistical test(s) used AND whether they are one- or two-sided<br>*Only common tests should be described solely by name; describe more complex techniques in the Methods section.* |
| ☐ | ☒ | A description of all covariates tested |
| ☐ | ☒ | A description of any assumptions or corrections, such as tests of normality and adjustment for multiple comparisons |
| ☐ | ☒ | A full description of the statistical parameters including central tendency (e.g. means) or other basic estimates (e.g. regression coefficient) AND variation (e.g. standard deviation) or associated estimates of uncertainty (e.g. confidence intervals) |
| ☐ | ☒ | For null hypothesis testing, the test statistic (e.g. *F*, *t*, *r*) with confidence intervals, effect sizes, degrees of freedom and *P* value noted<br>*Give P values as exact values whenever suitable.* |
| ☒ | ☐ | For Bayesian analysis, information on the choice of priors and Markov chain Monte Carlo settings |
| ☒ | ☐ | For hierarchical and complex designs, identification of the appropriate level for tests and full reporting of outcomes |
| ☒ | ☐ | Estimates of effect sizes (e.g. Cohen's *d*, Pearson's *r*), indicating how they were calculated |

*Our web collection on statistics for biologists contains articles on many of the points above.*

## Software and code

Policy information about availability of computer code

| | |
|---|---|
| Data collection | Retrospective chart review. Data collected in RedCap. |
| Data analysis | N/A |

For manuscripts utilizing custom algorithms or software that are central to the research but not yet described in published literature, software must be made available to editors and reviewers. We strongly encourage code deposition in a community repository (e.g. GitHub). See the Nature Portfolio guidelines for submitting code & software for further information.

## Data

Policy information about availability of data

All manuscripts must include a data availability statement. This statement should provide the following information, where applicable:

- Accession codes, unique identifiers, or web links for publicly available datasets
- A description of any restrictions on data availability
- For clinical datasets or third party data, please ensure that the statement adheres to our policy

The data that support the findings of this study are available from the corresponding author upon reasonable request.

# Human research participants

Policy information about studies involving human research participants and Sex and Gender in Research.

| | |
|---|---|
| Reporting on sex and gender | 96.6% of patients were female (Table 1); information about gender was not collected. |
| Population characteristics | Details are provided in Table 1. |
| Recruitment | Patients with early breast cancer who underwent surgery at DFCI were offered to participate in the COQD registry |
| Ethics oversight | Dana-Farber IRB |

Note that full information on the approval of the study protocol must also be provided in the manuscript.

# Field-specific reporting

Please select the one below that is the best fit for your research. If you are not sure, read the appropriate sections before making your selection.

☒ Life sciences ☐ Behavioural & social sciences ☐ Ecological, evolutionary & environmental sciences

For a reference copy of the document with all sections, see nature.com/documents/nr-reporting-summary-flat.pdf

# Life sciences study design

All studies must disclose on these points even when the disclosure is negative.

| | |
|---|---|
| Sample size | Convenience sample size - all patients who signed consent to participate in our registry. |
| Data exclusions | N/A |
| Replication | N/A |
| Randomization | N/A |
| Blinding | N/A |

# Reporting for specific materials, systems and methods

We require information from authors about some types of materials, experimental systems and methods used in many studies. Here, indicate whether each material, system or method listed is relevant to your study. If you are not sure if a list item applies to your research, read the appropriate section before selecting a response.

## Materials & experimental systems

| n/a | Involved in the study | |
|---|---|---|
| ☒ | ☐ | Antibodies |
| ☒ | ☐ | Eukaryotic cell lines |
| ☒ | ☐ | Palaeontology and archaeology |
| ☒ | ☐ | Animals and other organisms |
| ☐ | ☒ | Clinical data |
| ☒ | ☐ | Dual use research of concern |

## Methods

| n/a | Involved in the study | |
|---|---|---|
| ☒ | ☐ | ChIP-seq |
| ☒ | ☐ | Flow cytometry |
| ☒ | ☐ | MRI-based neuroimaging |

## Clinical data

Policy information about clinical studies

All manuscripts should comply with the ICMJE guidelines for publication of clinical research and a completed CONSORT checklist must be included with all submissions.

| | |
|---|---|
| Clinical trial registration | N/A - not a clinical trial |
| Study protocol | N/A |
| Data collection | Clinicopathologic and genetic data from all consecutive patients with gBRCA1/2 PV who underwent surgery between 1/4/2016 and |

| | |
|---|---|
| Data collection | 4/7/2022 for a first diagnosis of HER2-negative invasive breast cancer at Dana-Farber Brigham Cancer Center between 2016 and 2022 were extracted from prospectively collected institutional datasets. |
| Outcomes | Eligibility for adjuvant olaparib was defined according to the OlympiA study inclusion criteria. Clinicopathological caracteristics and treatment patterns were compared between eligible and ineligible patients using Fisher's exact test, Chi-squared test, or Wilcoxon rank-sum test, as appropriate. iDFS and RFS were defined according to STEEP 2.0 criteria and calculated using the Kaplan-Meier method with the log-rank test to compare between eligible and ineligible patients. As exploratory analyses, the proportion of gBRCA PV carriers with HR+ breast cancer deemed as high-risk by the monarchE criteria (1-3 positive nodes and grade 3 or tumor size>5 cm) and the overlap between eligibility to OlympiA and monarchE were assessed. |
